# Spin Isoenergetic Process and the Lindblad Equation

**DOI:** 10.3390/e21050503

**Published:** 2019-05-17

**Authors:** Congjie Ou, Yuho Yokoi, Sumiyoshi Abe

**Affiliations:** 1 Physics Division, College of Information Science and Engineering, Huaqiao University, Xiamen 361021, China; 2 Department of Physical Engineering, Mie University, Mie 514-8507, Japan; 3 Institute of Physics, Kazan Federal University, 420008 Kazan, Russia; 4 ESIEA, 9 Rue Vesale, 75005 Paris, France

**Keywords:** quantum thermodynamics of spin, weak invariants, isoenergetic process, Lindblad equation

## Abstract

A general comment is made on the existence of various baths in quantum thermodynamics, and a brief explanation is presented about the concept of weak invariants. Then, the isoenergetic process is studied for a spin in a magnetic field that slowly varies in time. In the Markovian approximation, the corresponding Lindbladian operators are constructed without recourse to detailed information about the coupling of the subsystem with the environment called the energy bath. The entropy production rate under the resulting Lindblad equation is shown to be positive. The leading-order expressions of the power output and work done along the isoenergetic process are obtained.

## 1. Introduction

Besides its importance for nanoscience, nanotechnology and quantum engineering in general, quantum thermodynamics has a particular status in contemporary physics. It sheds fresh light on both classical and quantum theories. For instance, the quantum-mechanical violation of the law of equipartition of energy seems to require careful reexaminations of even the isothermal process of the ideal gas. It is also the case for the connection between the adiabatic and isoentropic processes. Classical mechanics is described by a set of dynamical variables, whereas quantum mechanics contains not only dynamical variables but also the Hilbert space. Accordingly, quantum mechanics may lead to novel concepts that have no counterparts in classical thermodynamics. The dephasing bath [1] and energy bath [2,3] can be thought of as typical such examples. Therefore, it is of significance to elucidate the implications of such baths to thermodynamics.

In the present paper, we develop a discussion about the isoenergetic process associated with the energy bath within the framework of finite-time quantum thermodynamics. We employ a single Pauli spin in a time-dependent magnetic field and describe its nonequilibrium subdynamics based on the Lindblad equation [4,5], which is known to be unique in the Markovian approximation. It is shown how the Lindbladian operators can be determined without detailed information about the interaction between the subsystem and the energy bath. The resulting subdynamics is seen to be unital [6] and accordingly has a non-negative entropy production rate. The power output and work done are evaluated in the leading order.

The paper is organized as follows. In Section 2, a succinct explanation is presented about the concept of weak invariants, which is directly connected to the isoenergetic process. In Section 3, the Lindbladian operators are determined for the Pauli spin in a time-dependent magnetic field, and the entropy production rate under the Lindblad equation thus obtained is discussed. Then, based on the result in Section 3, finite-time thermodynamics of the power output and work in the leading order is studied in Section 4. Section 5 is devoted to concluding remarks.

## 2. Weak Invariant and Isoenergetic Process

The concept of weak invariants has wide universality. Let us consider a master equation

(1) $$i\;\hbar \frac{\partial \;\rho }{\partial \;t}=£(\rho )$$

for the density matrix ρ describing the state of a quantum subsystem, where £ is a certain linear superoperator. Henceforth, ℏ is set equal to unity for the sake of simplicity. Then, a weak invariant I=I(t) associated with this master equation is defined as a solution of the following equation [7]:

(2) $$i\frac{\partial I}{\partial t}\;+\;{£}^{*}\;(I)\;=\;0.$$

In this equation, £^*^ stands for the adjoint of £ defined by tr (*Q* £(*R*)) = tr(£^*^(*Q*) *R*), provided that *Q* £(*R*) and therefore also £^*^(*Q*) *R*] should be trace-class. Then, it follows from Equations (1) and (2) that the expectation value of *I* is conserved:

(3) $$\frac{d\langle I\rangle }{dt}=0$$

with the notation 〈Q〉≡tr(Qρ), although the spectrum of *I* is time-dependent, in general.

Our purpose is to clarify the implication of the energy bath to quantum thermodynamics. For it, what to be contemplated is the isoenergetic processes, along which the internal energy of the subsystem is kept constant through energy transfer between the subsystem and the energy bath. A point is that the energy transfer that may be realized not in the form of heat but rather in the dynamical manner [8,9].

The isoenergetic processes have been discussed for a “three-stroke” engine [3,10], in which however no explicit time evolution of the subsystem has been considered. In view of finite-time quantum thermodynamics, a density matrix evolves in time according to a master equation. Here, let us assume Markovianity of the subdynamics. In this case, the superoperator in Equation (1) has the Lindblad form [4,5]:

(4) $$£(\rho )=\left[H(t),\;\rho \right]-\frac{i}{2}\sum_{i}a_{\;i}\left(L_{\;i}^{\;†}\;L_{i}\;\rho +\rho \;L_{\;i}^{\;†}\;L_{i}-2L_{i}\rho L_{\;i}^{\;†}\right),$$

where H(t) is the time-dependent Hamiltonian and $a_{\;i}$’s are nonnegative *c*-number coefficients. Both the coefficients and the Lindbladian operators $L_{\;i}$’s may also depend on time, explicitly.

The weak invariant relevant to the isoenergetic process is the time-dependent Hamiltonian itself. Thus, the internal energy

(5) U=tr(H(t) ρ)

is required to be conserved under time evolution generated by the Lindblad equation. Such a condition is fulfilled if the Hamiltonian obeys the following equation:

(6) $$\frac{\partial \;H(t)}{\partial \;t}-\frac{1}{2}\sum_{i}a_{\;i}\left(L_{\;i}^{\;†}\;L_{i}\;H(t)+H(t)\;L_{\;i}^{\;†}\;L_{i}-2L_{\;i}^{\;†}\;H(t)\;L_{i}\right)=0,$$

which comes from Equation (2) with Equation (4).

In general, it is a nontrivial task to determine the Lindbladian operators in Equations (4) and (6), since it needs detailed knowledge of how the subsystem interacts with the environment. However, Equation (6) tends to make it possible to determine them without such knowledge [11].

Closing this section, we make some comments on the concept of weak invariants. Firstly, it has recently been discovered [12] that there exists a connection between weak invariants and the action principle for corresponding master equations. Secondly, such quantities clearly have their classical analogs. For instance, a weak invariant associated with the classical Fokker-Planck equation is discussed in Reference [13]. Thirdly, it is worth mentioning that the Lindblad equation with a time-dependent Hamiltonian can describe the dynamics toward an “instantaneous attractor” in the nonadiabatic regime [14], inside which isoenergeticity is realized.

## 3. Weak Invariant of Spin in Time-Dependent Magnetic Field and Lindbladian Operators

In recent years, quantum thermodynamics of a two-level system has been discussed in the literature [15,16]. Here, we consider a single spin in a time-dependent magnetic field B(t). The Hamiltonian reads

(7) H(t)=B(t)⋅σ

with σ being the Pauli-matrix vector. The constant involving the gyromagnetic ratio is set equal to unity. In the thermodynamic context, variation of the magnetic field should be slow. That is, the time scale of the variation is much longer than those of relaxation and quantum dynamics.

From the linearity of Equation (6) with respect to the Hamiltonian and the su(2) Lie algebra of the spin, it is natural to examine the following Lindbladian operators:

(8) $$L_{\;i}=\sigma_{i}\;(i=1,\;2,\;3).$$

These are Hermitian, and accordingly the subdynamics is unital [6]. Substituting Equation (8) into Equation (6) and using linear independence of the Pauli matrices, we have

(9) $$\dot{B}(t)=-2\;A\;B(t)+2c\;B(t),$$

where the overdot denotes differentiation with respect to time, and *A* and *c* are given by

(10) $$A=\left(\begin{array}{ccc} a_{1} & 0 & 0\\ 0 & a_{2} & 0\\ 0 & 0 & a_{3} \end{array}\right),$$

(11) $$c=\mathrm{tr}\;A=a_{1}+a_{2}+a_{3},$$

respectively. From Equation (9), we see that

(12) $$B(t)\cdot \dot{B}(t)=2\left(a_{2}+a_{3}\right)\;B_{1}^{\;2}(t)+2\left(a_{3}+a_{1}\right)\;B_{2}^{\;2}(t)+2\left(a_{1}+a_{2}\right)\;B_{3}^{\;2}(t),$$

which is positive since $a_{\;i}$’s are nonnegative and not all of them can be zero. Therefore, the magnitude B(t)=|B(t)| has to monotonically increase in time. In addition, from Equation (9), we find the coefficients to be given by

(13) $$a_{\;1}=\frac{1}{4}\left(-\frac{{\dot{B}}_{\;1}}{B_{\;1}}+\frac{{\dot{B}}_{\;2}}{B_{\;2}}+\frac{{\dot{B}}_{\;3}}{B_{\;3}}\right),\;a_{\;2}=\frac{1}{4}\left(\frac{{\dot{B}}_{\;1}}{B_{\;1}}-\frac{{\dot{B}}_{\;2}}{B_{\;2}}+\frac{{\dot{B}}_{\;3}}{B_{\;3}}\right),\;a_{\;3}=\frac{1}{4}\left(\frac{{\dot{B}}_{\;1}}{B_{\;1}}+\frac{{\dot{B}}_{\;2}}{B_{\;2}}-\frac{{\dot{B}}_{\;3}}{B_{\;3}}\right).$$

Thus, without detailed information about the interaction between the subsystem and the environment, the explicit form of the Lindblad equation is now fully determined as follows:

(14) $$i\;\frac{\partial \;\rho }{\partial \;t}=\left[H(t),\;\rho \right]-\frac{i}{2}\sum_{i=1}^{3}\;a_{\;i}\left[\sigma_{i},\;\left[\sigma_{i},\;\rho \right]\right].$$

We note that the second term on the right-hand side, termed the dissipator, is small and is of the order ${\dot{B}}_{\;i}(t)$’s. Equation (14) belongs to a class of finite-level systems in contact with a singular reservoir consisting of particles with the vanishing correlation time [17]. On the other hand, what is peculiar here is highlighted in Equation (13) that purely comes from isoenergeticity, without recourse to the detailed property of the energy bath.

Hermiticity of the Lindbladian operators in Equation (8), or equivalently the fact that the subdynamics is unital, can have a significant implication for the isoenergetic process. As shown in Reference [18] (see, also Reference [11] and a discussion generalized to the Rényi entropy in Reference [19]), time evolution of the von Neumann entropy

(15) S [ρ]=−tr (ρlnρ)

under the Lindblad equation in its general form in Equation (1) with Equation (4) satisfies

(16) $$\frac{d\;S}{d\;t}=\sum_{i}\;a_{\;i}\;\Gamma_{\;i},$$

(17) $$\Gamma_{\;i}\geq \langle \left[L_{\;i}^{\;†},\;L_{i}\right]\rangle ,$$

where the Boltzmann constant is set equal to unity. Since the Lindbladian operators in the present case are Hermitian, the subdynamics tends to produce the entropy in time,

(18) $$\frac{d\;S}{d\;t}\geq 0,$$

as desired.

## 4. Temporally-Local Equilibrium State, Power Output and Work along Isoenergetic Process

A nonequilibrium thermodynamic system with a slowly-varying Hamiltonian is dominantly described by a locally-equilibrium state, in which the thermodynamic variables also vary slowly. In the present case, what is relevant is the temporally-local equilibrium state given by

(19) $$\rho^{\;(0)}=\frac{1}{Z\;(t)}\exp\;\left(-\beta (t)H(t)\right),$$

where Z(t) is the partition function

(20) Z(t)=tr exp(−β(t) H(t)).

β(t) is the time-dependent inverse temperature that slowly varies in time [see Equation (26) below].

For the Hamiltonian in Equation (7), the explicit expression of Equation (19) is the familiar one

(21) $$\rho^{\;(0)}=\frac{1}{2}\left\{I-\left(n(t)\cdot \sigma \right)\tanh\left(\beta (t)\;B(t)\right)\right\},$$

where *I* is the 2 × 2 unit matrix and n(t) is a unit vector defined by n(t)=B(t)/B(t).

The full density matrix may be expanded around the state in Equation (21) as follows:

(22) $$\rho =\rho^{\left(0\right)}+\rho^{\left(1\right)}+\cdot \cdot \cdot ,$$

where the expansion should be performed in terms of the elements of $\dot{B}(t)$ and its higher-order derivatives. It is natural to assume that all of the correction terms are traceless. Substituting Equation (22) into Equation (14), the first-order correction is found to satisfy

(23) $$\left[H(t),\;\rho^{\left(1\right)}\right]=i\;\frac{\partial \;\rho^{\left(0\right)}}{\partial \;t}+\frac{i}{2}\sum_{i=1}^{3}a_{\;i}\;\left[\sigma_{i},\;\left[\sigma_{i},\;\rho^{\left(0\right)}\right]\right],$$

for instance.

In accordance with Equation (22), the internal energy is also expanded as follows:

(24) $$U=U^{\left(0\right)}+U^{\left(1\right)}+\cdot \cdot \cdot .$$

The isoenergeticity condition should be satisfied in each order. For the leading-order term

(25) $$U^{\left(0\right)}=\mathrm{tr}\left(H(t)\;\rho^{\;(0)}\right)=-B(t)\;\tanh\left(\beta (t)\;B(t)\right),$$

we find that the isoenergeticity condition, $dU^{\left(0\right)}/d\;t=0$, in the leading order gives rise to

(26) $$\dot{B}(t)\;\left[\sinh\left(2\beta (t)\;B(t)\right)+2\beta (t)\;B(t)\right]=-2B^{\;2}(t)\;\dot{\beta }(t).$$

Since $\dot{B}(t)$ is positive due to Equation (12) resulting from the Lindblad equation, $\dot{\beta }(t)$ is necessarily negative, implying that the temperature monotonically increases in time.

Now, let us discuss the power output defined by

(27) $$P(t)=-\mathrm{tr}\;\left(\dot{H}(t)\;\rho \right).$$

The leading-order contribution comes from the temporally-local equilibrium state and is calculated to be

(28) $$P(t)=-\;\dot{B}(t)\cdot \mathrm{tr}\;\left(\sigma \;\rho^{\left(0\right)}\right)=\dot{B}(t)\cdot n(t)\;\tanh\left(\beta (t)\;B(t)\right).$$

An interesting point is that, similarly to the case of the time-dependent harmonic oscillator [20], this quantity can be expressed in terms of the internal energy as follows:

(29) $$P(t)=-\frac{\dot{B}(t)}{B(t)}\;U^{\;(0)}.$$

From this expression, the work done during the time interval $t_{\;i}\leq t\leq t_{f}$ along the isoenergetic process is given, in the leading order, by

(30) $$W=\int_{t_{i}}^{t_{f}}dt\;P(t)=-\;U^{\;(0)}(t_{i})\;\int_{t_{i}}^{t_{f}}dt\;\frac{\dot{B}(t)}{B(t)}=B(t_{i})\tanh\left(\beta (t_{i})\;B(t_{i})\right)\;\ln\left[\frac{B(t_{f})}{B(t_{i})}\right],$$

where the initial value is used for the conserved internal energy.

## 5. Concluding Remarks

Toward clarifications of the physical properties of exotic baths present in quantum thermodynamics but absent in classical thermodynamics, we have focused our attention on the isoenergetic process that is connected with the energy bath. For this purpose, we have studied a single spin in a magnetic field slowly varying in time based on the Lindblad equation. We have shown how the isoenergeticity condition can determine the Lindbladian operators without recourse to detailed knowledge of the interaction between the subsystem and the energy bath. We have developed a discussion about finite-time thermodynamics of such a system and evaluated the power output and the work in the leading order.

The result given in Equation (30) implies that the work is determined only by the initial and final values of the variables without depending on paths of the magnetic field in the parameter **B**-space. This would be a common feature of the isoenergetic processes in the leading order.

In the present work, we have treated a single Pauli spin. As known, by virtue of the structure of spin algebra, any density matrix of a multispin system can be expanded in terms of the spin matrices, their tensor products as well as the unit matrix. Therefore, the temporally-local equilibrium state in Equation (21) can be generalized to the multispin case [21].

Here, we have considered only the energy bath, which is nothing but one of the exotic baths in quantum thermodynamics. Existence of various baths indicates that there may be ensemble-dependent dynamics. In this point, it is worth mentioning that such dynamics are also suggested in nanothermodynamics of classical small systems, in which fluctuations can be very large [22,23]. Much is yet to be clarified about the quantum-classical correspondence in thermodynamics.
